# BALB/c Mice Can Learn Touchscreen Visual Discrimination and Reversal Tasks Faster than C57BL/6 Mice

**DOI:** 10.3389/fnbeh.2017.00016

**Published:** 2017-01-31

**Authors:** Karly M. Turner, Christopher G. Simpson, Thomas H. J. Burne

**Affiliations:** ^1^Queensland Brain Institute, The University of QueenslandSt Lucia, QLD, Australia; ^2^Queensland Centre for Mental Health Research, The Park Centre for Mental HealthRichlands, QLD, Australia

**Keywords:** visual discrimination, reversal learning, touchscreen, C57BL/6, BALB/c

## Abstract

Touchscreen technology is increasingly being used to characterize cognitive performance in rodent models of neuropsychiatric disorders. Researchers are attracted to the automated system and translational potential for touchscreen-based tasks. However, training time is extensive and some mouse strains have struggled to learn touchscreen tasks. Here we compared the performance of commonly used C57BL/6 mice against the BALB/c mice, which are considered a poor performing strain, using a touchscreen task. BALB/c and C57BL/6 mice were trained to operate the touchscreens before learning a visual discrimination (VD) and reversal task. Following touchscreen testing, these strains were assessed for differences in locomotion and learned helplessness. BALB/c mice finished training in nearly half the number of sessions taken by C57BL/6 mice. Following training, mice learned a VD task where BALB/c mice again reached criteria in fewer than half the sessions required for C57BL/6 mice. Once acquired, there were no strain differences in % correct responses, correction trials or response latency. BALB/c mice also learnt the reversal task in significantly fewer sessions than C57BL/6 mice. On the open field test C57BL/6 mice traveled further and spent more time in the center, and spent less time immobile than BALB/c mice on the forced swim test (FST). After touchscreen testing, strains exhibited well-established behavioral traits demonstrating the extensive training and handling from touchscreen testing did not alter their behavioral phenotype. These results suggest that BALB/c mice can be examined using touchscreen tasks and that task adaptations may improve feasibility for researchers using different strains.

## Introduction

Touchscreen testing in rodents has become widespread over the last decade with a focus on automated testing of cognitive domains relevant to neuropsychiatric illness. These studies have explored the neurobiology of task performance, the effects of pharmacological agents and cognitive deficits in animal models (Chudasama and Robbins, 2003; Brigman et al., 2010; Talpos et al., 2012). Although operant experiments have traditionally favored rats, touchscreen technology has provided researchers a new tool for examining cognition in mice (Bussey et al., 2001; Brigman et al., 2010; Young et al., 2013). Furthermore, the protocols available reflect features of tasks used in monkeys and humans (Horner et al., 2013; Hvoslef-Eide et al., 2015). This has promoted translational studies exploring cognition across species (Bussey et al., 2012; Nithianantharajah et al., 2013). Two paradigms commonly used across species, in both traditional and touchscreen assays, are discrimination and reversal learning (Izquierdo and Jentsch, 2012; Young et al., 2013; Hvoslef-Eide et al., 2015). Discrimination learning provides a measure of perceptual ability and associative learning potential (Horner et al., 2013). On the other hand, reversal learning has been used to examine behavioral flexibility; an aspect of cognitive functioning that is disrupted in a range of neuropsychiatric disorders (Brigman et al., 2010).

Cognitive performance in both rats and mice has been shown to be highly strain dependent (Didriksen and Christensen, 1993; Turner and Burne, 2013; Graybeal et al., 2014; Kim et al., 2015; Kumar et al., 2015). C57BL/6 mice are a popular strain for behavioral and genetic studies, and have been used as a standard strain against which others are compared (Crawley et al., 1997; Kalueff and Tuohimaa, 2004; Izquierdo et al., 2006; Young et al., 2009; Harms et al., 2012). Meanwhile, BALB/c mice often display poor learning and cognitive performance compared to other strains (Crawley et al., 1997; Van Dam et al., 2006; Graybeal et al., 2014). Recently, the BALB/c strain was shown to be “severely impaired” in basic training, visual discrimination (VD) and reversal learning using touchscreen chambers (Graybeal et al., 2014). In fact, large proportions of the BALB/c cohort failed to reach criteria on both these tasks even after 60 sessions (Graybeal et al., 2014). These results indicate that BALB/c mice may be an inappropriate strain to select for touchscreen experiments, however very few studies have assessed cognition in BALB/c mice using touchscreen tasks. Other albino strains also had poor performance and it has been shown that albino strains do not perform as well on visual tasks compared to motor-learning or olfactory tasks (Brown and Wong, 2007; Yeritsyan et al., 2012). In addition, BALB/c mice are known as a vulnerable, anxious and emotional strain (Belzung and Griebel, 2001; Brooks et al., 2005; Brinks et al., 2007; Razzoli et al., 2011). It is well known that stress influences cognition (Joëls and Baram, 2009) and therefore it is plausible that BALB/c mice do not have cognitive deficits, but that their poor performance has been confounded by their anxious phenotype.

Task optimization has been conducted for touchscreen performance in rats (Bussey et al., 2008) and although protocol suggestions have been made (Horner et al., 2013; Mar et al., 2013), an equivalent study has not been published for mice. It cannot be assumed that the same parameters will equally suit mice and rats, because there are significant differences in preferences and performance traits between these species (Crawley, 1999; Young et al., 2013). For example, mice appear to prefer nose poking whereas rats will readily lever press (Crawley, 2007). Furthermore, optimal parameters may differ based on strain-specific behavioral traits. Although separately testing all elements of the training regime would be quite an onerous study, small adaptations of task conditions are common between experimental sites (for example pellet or liquid rewards). The aim of this study was to compare performance of C57BL/6 and BALB/c mouse on touchscreen VD and reversal tasks with minor protocol modifications. The goal was to demonstrate the anxious and poor learning BALB/c mouse could perform as well as C57BL/6 mice.

## Materials and Methods

### Animals

Male 3-month old BALB/cARC (*N* = 12) and C57BL/6JARC (*N* = 12) mice were obtained from the Animal Resources Centre (Murdoch, WA, Australia) and housed in groups of four in individually ventilated caging (OptiMICE, Animal Care Systems, Centennial, CO, USA) with bedding on 12-h light cycle (lights on at 0700) in a room maintained at 21 ± 1°C and 50 ± 10% humidity. Individual tail markings were used to identify animals throughout the experiment. Prior to training and during testing mice were food-restricted to 90% of their free-feeding body weight. This was conducted by adjusting the amount of food provided on a daily basis depending on their % free-feeding weight and in small enough pieces to ensure all mice were able to consume the food without fighting. Food was provided at the same time each day with *ad libitum* access to water in the home cage. All procedures were performed with the approval from The University of Queensland Animal Ethics Committee, under the guidelines of the National Health and Medical Research Council of Australia.

### Touchscreen Apparatus

Testing was conducted in commercially available operant chambers (Bussey Mouse Touchscreen Chamber, Campden Instruments, UK) equipped with a touchscreen, house light, reward dispenser and magazine. Each operant chamber was contained within a sound-attenuating box, fitted with an overhead camera to monitor and record sessions. Infrared beams on the surface of the touchscreen and inside the magazine were used to determine responses. A black 2-window (7.0 × 7.5 cm) mask was placed in front of the touchscreen to promote responding to the correct locations. Mice were brought into the dimly lit (10% fluorescent lighting) testing room to habituate for a minimum of 30 min prior to each training session as testing was conducted with the house light off. Protocol operation, including stimulus presentation and reward delivery, and data analysis was conducted by ABET II Touch software (Campden Instruments, UK). The mask, touchscreen, grid floor and tray were cleaned with 70% ethanol between animals. At the end of the day, all reward lines were flushed with warm water then pumped dry to prevent blockages.

### Touchscreen Training

Following the recommendations of Horner et al. (2013), mice were first trained to use the touchscreens, then tested on VD and reversal.

Although others have used food pellet rewards, this may limit the number of trials per session that a mouse will complete (Horner et al., 2013), therefore a liquid reward was used in this study. To acclimate mice to the reward, a small quantity of strawberry milk (Breaka, Parmalat, South Brisbane, QLD, Australia) was provided in their home cage in a 50 ml falcon tube lid (BD Biosciences) for 3 days prior to commencement of training. Mice were habituated to the testing room by bringing their home cage into the testing room for a minimum of 30 min prior to each session. Mice then progressed through the stages of training as outlined by previous studies with some modifications (Horner et al., 2013). Changes included removing the auditory correct tone, changing the reward from pellet to liquid, increasing the number of trials within a session, and reducing the inter-trial interval (ITI) to 5 s. These steps were undertaken to: (a) increase the pace of the task to reduce lag time between trials; (b) decrease distractibility from neighboring chamber auditory cues; and (c) increase the amount of training events per session. Removing sounds was also incorporated to reduce additional anxiety in the BALB/c mice. The images were also different from previous reports. They were selected for symmetry and a lack of straight edges, as angled lines were to be used as stimuli in PAL studies with these mice.

First, mice were trained on the Habituation stage, which involved learning to collect a reward when the tray illuminated, and they were required to complete 50 trials within 20 min on two consecutive sessions. Then they moved to Initial Touch, where they learnt to touch the stimuli (white square) on the screen to receive a 3× reward or after 30 s of no activity they received a single reward. Mice were progressed to Must Touch Stimuli after completing 100 trials within 30 min or after a maximum of five sessions on Initial Touch. Mice were now required to touch the stimuli to receive a reward and again complete 100 trials in under 30 min. Following this step, mice moved to Must Initiate where they were required to enter the reward tray to start the trial and they were now required to complete 100 trials in 60 min. Then finally mice moved to Punish Incorrect where a time-out occurred if they touched the incorrect location and they were required to complete 100 trials in under 60 min with at least 70% correct. Except for the changes outlined here, the details of the pre-training steps were as previously reported (Horner et al., 2013). After completing training mice moved to the VD task.

### Visual Discrimination (VD) Task

**Figure 1:**
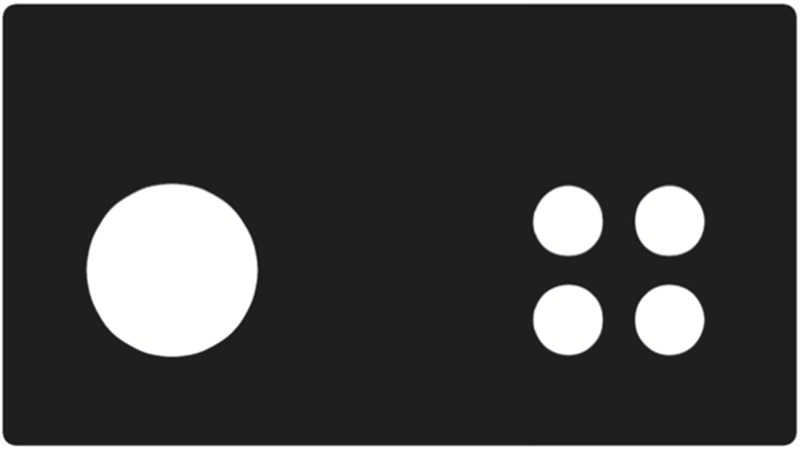
**The two circular stimuli used for visual discrimination (VD) tasks.** Mice were trained to respond to one stimulus irrespective of the presentation location and this was counter balanced across the cohort. The reversal task required the mouse to stop responding to the previously rewarded stimulus and to respond to the alternative image to receive rewards.

The VD task required rodents to discriminate between two visual stimuli and to learn which stimulus was associated with a reward. Within each trial, two stimuli were presented and if the correct one was selected the mouse received a reward. For this task the two images used were a single large white circle and four small white circles which were selected as they have similar features, similar luminance levels and do not differ in orientation aspects (Figure 1). The image that was rewarded was counter balanced within each group but remained constant for individual mice. Mice were required to complete 100 trials in under 60 min and achieve at least 80% correct. Correction trials are critical to this protocol to prevent mice simply selecting one window, which would be rewarded on 50% of trials. If they chose the wrong window, the image would be repeated in that same window until the mouse selected the correct image, therefore a lose-shift approach would be beneficial to learning this task. Once this task was acquired, the contingency was switched for reversal so the previously unrewarded stimulus must now be selected and the previously rewarded stimulus is now incorrect. The rate of acquisition of the new pairing provides a measure of behavioral flexibility and testing was conducted until reaching the criteria of 80% correct or a maximum of 20 sessions.

### Touchscreen Measures

There are many outcome measures that can be generated from operant testing. For the tasks used in this study these included the number of trials completed, session duration, the number of response type (correct, incorrect, blank), response latency, reward latency, omissions (failure to respond), irrelevant touches (during ITI or time out), front and rear beam breaks, and the number of sessions required to reach criteria. Within the touchscreen protocols, if a mouse selects the incorrect window the trial was repeated with the same stimuli and locations as a correction trial until the correct window was chosen. These correction trials prevent the development of a side bias by preventing reward until an alternative response was detected.

### Open Field

To measure locomotor activity mice were individually placed in square Perspex chambers equipped with an array of infrared beams to track body position (27 × 27 × 20 cm, Med Associates Inc., St. Albans, VT, USA). The chamber was housed within a ventilated sound attenuated box with white light-emitting diode (LED) lighting to maintain constant luminance of 18 lux. The chamber was cleaned with 70% ethanol between animals. The total distance traveled was calculated in 5 min time bins over the 30 min session. Med Associates Behavior Monitor software was also used to calculate the % time spent in the center of the arena (18 × 18 cm^2^), manufacturer specifications, over the 30 min session.

### Forced Swim Test

The forced swim test (FST) is commonly used to measure behavioral despair. Mice were individually placed into a clear cylindrical vessel (13 cm diameter × 20 cm high) containing clean 25°C water to a depth of 11.5 cm. At this depth, mice cannot touch the bottom or escape from the top. Mice were placed into the water for 10 min and activity was recorded with an overhead video camera (CCD Mini CCIR, Samsung). At the end of testing mice were allowed to dry in a cage filled with paper towel prior to returning to their home cage. Videos were then analyzed using tracking software (Ethovision XT 9, Noldus, Netherlands) to measure the total distance traveled and the total duration of immobility (defined as 0%–5% activity) in 1 min time bins. Clean water was used for each animal and the container was wiped down with 70% ethanol.

### Statistical Analysis

Results were analyzed using SPSS software (version 20, SPSS Inc., Chicago, IL, USA). The main effect of strain on behavioral outcomes from the touchscreen and behavioral testing was subjected to independent *t*-tests, analysis of variance (ANOVA) or repeated measures ANOVA where required. Bonferroni correction was applied to account for multiple comparisons. If a significant interaction was detected, independent *t*-tests were performed. Greenhouse-Geisser correction was used where appropriate for violation of sphericity. Data is presented as mean ± SEM and statistical significance was determined if *p* < 0.05. Two BALB/c mice were not tested on the final behavioral tests due to acute illness and therefore 10 BALB/c mice were included in the final two behavioral tests.

## Results

### Touchscreen Training

**Figure 2:**
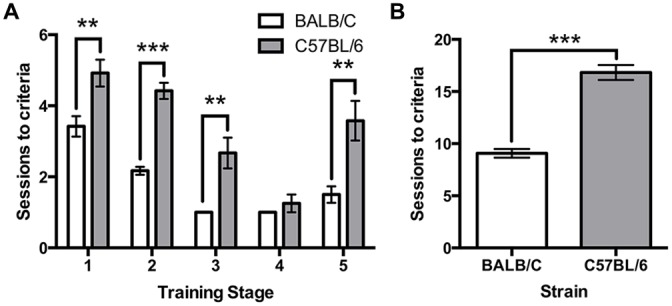
**Sessions required for touchscreen training. (A)** The number of sessions required to reach criteria was significantly greater for C57BL/6 mice compared to BALB/c mice on each training step except step 4. **(B)** Overall, this resulted in a greater number of sessions required to reach the final training step in C57BL/6 mice than in BALB/c mice. ***p < 0.001, **p < 0.01.

All mice were trained using the same criteria across five touchscreen training steps. A repeated measures ANOVA revealed a significant effect of strain (*F*_(1,22)_ = 87.52, *p* < 0.001) and a strain*training level interaction (*F*_(3.4,88)_ = 3.24, *p* = 0.022) on the number of sessions required to reach criteria. Independent *t*-tests found that C57BL/6 mice took significantly longer than BALB/c mice to acquire four of the five training steps (Habituation: (1) *t*_(22)_ = −3.16, *p* = 0.005; Initial Touch; (2), *t*_(16.0)_ = −8.82, *p* < 0.001; Must Touch; (3) *t*_(11.0)_ = −3.86, *p* = 0.003; Must Initiate; (4) *t*_(11.0)_ = −1.00, *ns*; Punish Incorrect; and (5) *t*_(14.7)_ = −3.46, *p* = 0.004; Figure 2A). Overall, the total number of sessions required to complete training was significantly greater for C57BL/6 mice than for BALB/c (*t*_(17.7)_ = −9.36, *p* < 0.001; Figure 2B).

### Visual Discrimination

**Figure 3:**
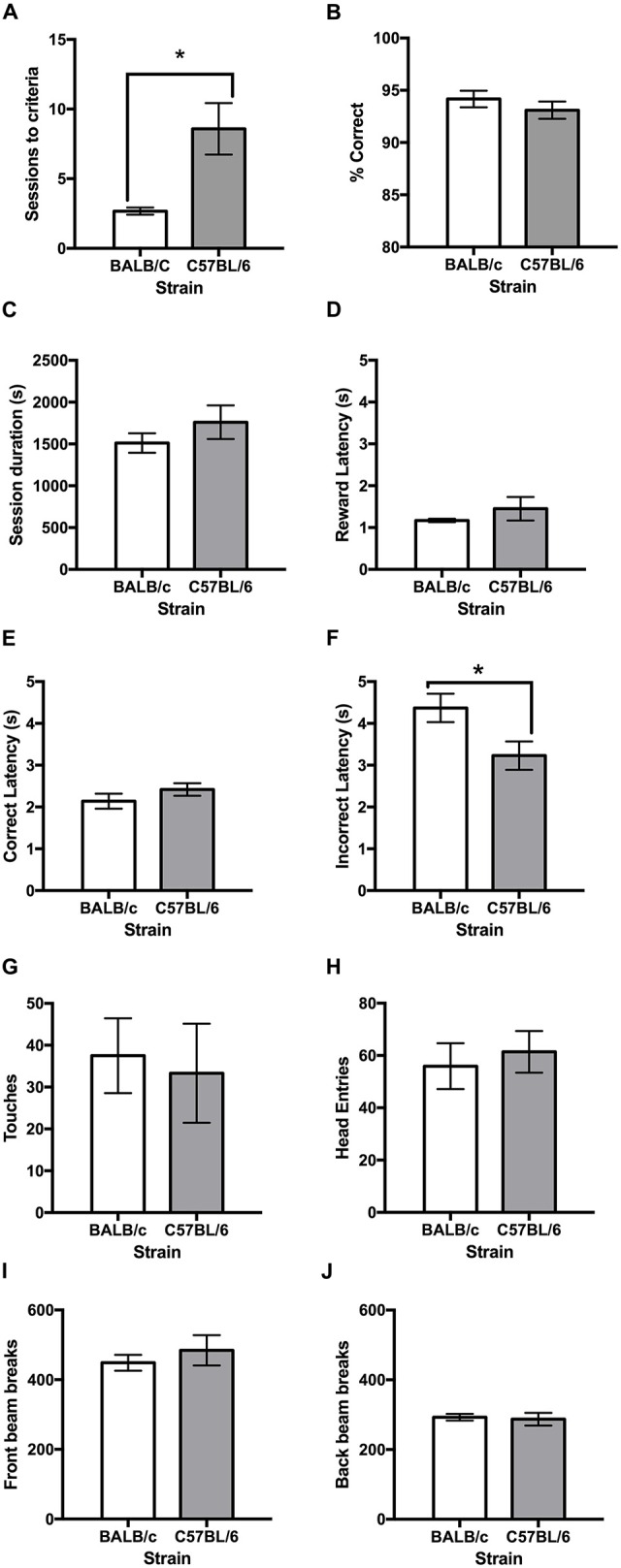
**Performance measures on the VD task for BALB/c and C57BL/6 mice on the final day of testing. (A)** It was found that BALB/c mice acquired the VD task in significantly fewer sessions than C57BL/6 mice. The strains did not differ on measures of **(B)** accuracy, **(C)** session duration, **(D)** reward latency or **(E)** correct latency. **(F)** However, BALB/c mice took longer that C57BL/6 mice to respond when incorrect; but did not differ on measures of **(G)** touches during inter-trial interval (ITI), **(H)** head entries during ITI, **(I)** front beam breaks or **(J)** back beam breaks. *p < 0.05.

**Figure 4:**
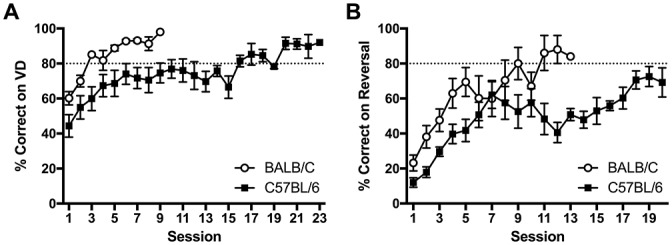
**Acquisition curves showing learning rate in BALB/c and C57BL/6 mice on (A)** VD and **(B)** reversal. Mice started near chance 50% on VD but all mice went on to reach criteria of >80% correct. Upon reversal % correct dropped to around 20% and five C57BL/6 mice did not acquire reversal within 20 sessions (white circles are BALB/c, black squares are C57BL/6).

Following training, both strains learnt the VD task and it was found that the C57BL/6 mice took more than twice as many sessions than the BALB/c mice to reach the criterion of 80% correct responses (*t*_(11.4)_ = −3.16, *p* = 0.009; Figures 3A, 4A). Once VD criteria of three sessions on >80% accuracy was obtained there was no significant difference between strains for accuracy, session length, reward latency, touches or head entries during the ITI or front/back beam breaks (Figure 3). However, BALB/c mice took significantly longer than C57BL/6 mice when they made an incorrect response (*t*_(20)_ = 2.35, *p* = 0.029; Figure 3F).

**Figure 5:**
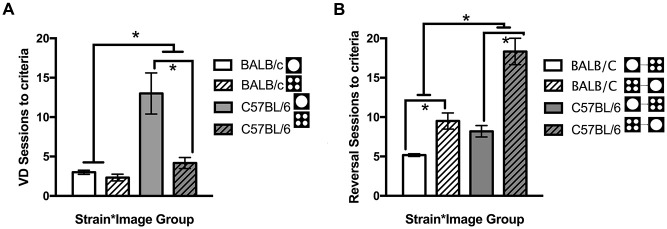
**Number of sessions required for mice to learn the VD and reversal tasks.** Overall BALB/c mice acquired VD and reversal in significantly fewer sessions than the C57BL/6 mice. **(A)** When VD was analyzed for image type C57BL/6 (but not BALB/c) mice learnt faster when responding to the four small circles compared to the single large circle. **(B)** Then on reversal, switching from the single large circle to the four small circles was acquired faster by both strains than the opposite switch from the four small circles to the single large circle (white bars are BALB/c, shaded bars are C57BL/6, open bars are mice originally trained on large circle, hatched bars are mice originally trained on four small circles), *p < 0.05.

In addition to comparing overall strain differences, performance was compared for each image used in VD. Across both strains, there were more sessions required to reach criteria for the single large circle compared to the four small circles (*t*_(12.3)_ = 2.36, *p* = 0.036). This result was primarily due to C57BL/6 mice where significantly fewer sessions were required when the four small circles were rewarded compared to those required to select the single large circle (*t*_(5.72)_ = −3.27, *p* = 0.018, Figure 5A). There was no significant difference between the images for BALB/c mice (*t*_(10)_ = 1.35, *ns*, Figure 5A). Mice were assigned to image groups pseudorandomly with no difference between image groups in the number of sessions required to learn the training steps (*t*_(22)_ = −0.135, *ns*).

After reaching criteria on the VD task, the contingency was reversed. Again C57BL/6 mice took significantly more sessions to reach criteria after reversal (*t*_(14.1)_ = 3.23, *p* = 0.006; Figure 4B). Reversal training ceased after 20 sessions at which point all 12 BALB/c mice had reached criteria, however five C57BL/6 mice did not reach criteria.

Furthermore it was found that reversal learning differed by image (*t*_(13.7)_ = −4.12, *p* = 0.001) with both BALB/c (*t*_(5.3)_ = −4.17, *p* = 0.008) and C57BL/6 (*t*_(9)_ = −5.17, *p* = 0.001) mice acquiring reversal in fewer sessions when they were initially trained to respond to the large circle and moved to the four small circles on reversal (Figure 5B). On the final session of testing there were no differences between Strains or Strain * Image interactions on any other measure, indicating deficits in task acquisition were not accompanied by changes in response latencies, addition touches, head entries or beam breaks in the chamber (Table 1).

**Table 1:** **Behavioral measures on the final session of reversal testing**.

	BALB/c		C57BL/6	
	Mean	±SEM	Mean	±SEM
Session length (s)	1506.2	109.65	1784.56	207.87
Trials completed	100	0	96.45	3.55
#Correction trials	13.42	2.93	21.55	7.14
% Correct	89.25	2.25	84.11	5.07
Left ITI touches	12.58	2.3	13.55	3.63
Right ITI touches	19.33	4.68	13.55	3.58
Total ITI touches	31.92	6.26	27.09	6.64
Total trials + CTs	113.42	2.93	118	5.66
Correct touch latency	1.85	0.11	2.3	0.25
Incorrect touch latency	3.1	0.47	3.44	0.35
Left correct touch latency	1.74	0.1	2.34	0.34
Right correct touch latency	1.95	0.19	2.26	0.25
Reward latency	1.19	0.03	1.17	0.05
Front beam breaks	445.92	20.24	467.91	51.97
Back beam breaks	301.58	20.17	302.45	17.62
Total head entries in ITI	69.5	12.77	65.73	10.54

There was no significant effect of Strain on any measure. When split for Strain * Image there were also no significant differences between groups.

### Open Field Test

**Figure 6:**
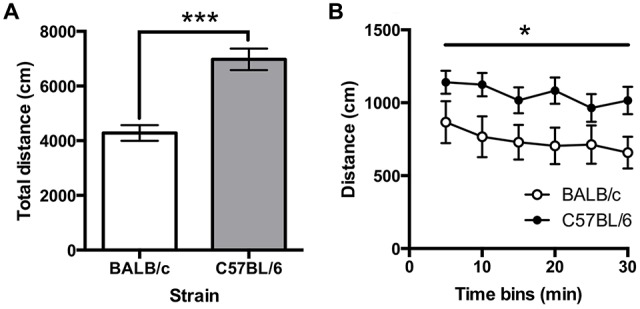
**Locomotor activity in the open field test. (A)** C57BL/6 (*N* = 12) mice moved significantly further than BALB/c (*N* = 10) mice and **(B)** this was consistent across the 30 min of testing (white bars and circles are BALB/c, shaded bars and circles are C57BL/6), ***p < 0.001, *p < 0.05.

Total distance traveled on the open field test was greater for C57BL/6 (*N* = 12) than for BALB/c (*N* = 10) mice (*t*_(20)_ = −5.30, *p* < 0.001; Figure 6A). A repeated measure ANOVA found that locomotion varied significantly across the 5 min time bins (*F*_(3.1,62.4)_ = 2.87, *p* = 0.018; Figure 6B) and there was a main effect of strain (*F*_(1,20)_ = 5.55, *p* = 0.029) with no strain * time interaction. These results indicated C57BL/6 mice moved significantly further than BALB/c mice across the session. It was also found that BALB/c mice spent significantly less time in the center of the open field (mean (±SEM) % center time 16.96 ± 2.80) compared to the C57BL/6 (48.28 ± 2.75; *t*_(20)_ = −5.30, *p* < 0.001).

### Forced Swim Test

**Figure 7:**
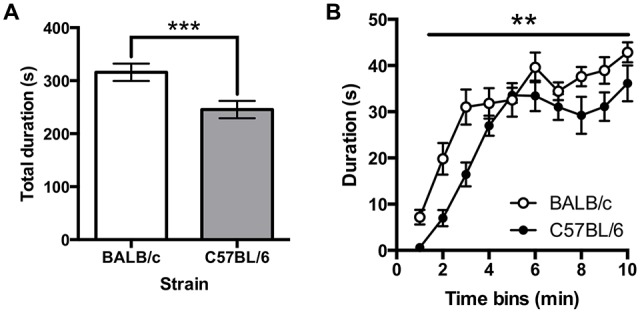
**Immobility in the forced swim test (FST). (A)** C57BL/6 (*N* = 12) mice spend significantly less time immobile than BALB/c (*N* = 10) mice over the 10 min test and **(B)** generally immobility increased across the test session, (white bars and circles are BALB/c, shaded bars and circles are C57BL/6), ***p < 0.001, **p < 0.01.

Immobility on the FST was greater in BALB/c (*N* = 10) compared to C57BL/6 (*N* = 12) mice (*t*_(20)_ = 3.18, *p* = 0.005; Figure 7A). A repeated measures ANOVA comparing immobility in 1 min time bins found that immobility significantly increased across the session (*F*_(6.3,180)_ = 41.86, *p* < 0.001; Figure 7B) and there was a main effect of strain (*F*_(1,20)_ = 10.10, *p* = 0.005) but there was no strain * time interaction. It was also found that total distance traveled was greater in C57BL/6 mice compared to BALB/c mice (*t*_(20)_ = −5.40, *p* < 0.001).

## Discussion

In this study we have demonstrated that BALB/c mice are capable of learning VD and reversal touchscreen tasks and at a significantly faster rate than C57BL/6 mice. These results are in contrast to previous reports of very poor performance of BALB/c mice when compared with the C57BL/6 and other inbred strains (Graybeal et al., 2014). Importantly, measures of behavioral despair and locomotion indicate that this cohort of mice has retained typical strain-specific behavioral traits.

Recently, a comprehensive assessment of performance was conducted for a range of inbred mouse strains on the touchscreen VD and reversal tasks (Graybeal et al., 2014). The strains differed in the number of sessions required to reach criteria on pre-training, discrimination and reversal learning. Specifically, C57BL/6 and BALB/c mice differed substantially on discrimination and reversal learning with 58% of BALB/c mice failing to discriminate stimuli and all of them failing to learn reversal after 60 sessions. In comparison, C57BL/6 mice reached criteria on VD in around 10 sessions and reversal after about 20 sessions. These values are similar to those obtained in our study and in earlier studies using C57BL/6 mice (Izquierdo et al., 2006). In contrast, the results of our study indicated BALB/c mice were not only capable of learning VD and reversal tasks, but that task acquisition was superior to C57BL/6 mice. These findings could be important for researchers using touchscreen chambers because the BALB/c strain provides a unique genetic and behavioral mouse model. In addition, as BALB/c mice may be difficult to assess using other cognitive platforms, these results indicate touchscreen testing may be a useful tool for researchers who already employ this strain.

Comparisons of C57BL/6 and BALB/c mice have produced mixed results on cognitive tasks. For example, BALB/c mice were not able to learn a VD swim tank task, whereas C57BL/6 mice successfully learnt both discrimination and reversal tasks (Brooks et al., 2005). However, BALB/c mice don’t typically perform well in swimming tasks (Brooks et al., 2005), which may generate strain-dependent effects related to anxiety rather than cognition. In contrast, BALB/c mice acquired and completed a modified holeboard task faster than C57BL/6 mice, however the strains performed similarly on reversal (Brinks et al., 2007). On an operant chaining task, BALB/c mice were found to be superior to C57BL/6 on a range of performance measures (Johnson et al., 2010). Due to the differences in sucrose consumption, it was suggested in this study that C57BL/6 mice satiated or habituated to the reward faster than BALB/c mice (Johnson et al., 2010). It is possible that BALB/c mice have a stronger preference for sweet rewards than C57BL/6. Although there was no significant difference in reward latency in this study, in a separate cohort we have found that BALB/c mice will consume significantly more strawberry milk than C57BL/6 mice when given free access, despite their lower body weight (unpublished data). The use of strawberry milk instead of grain pellets may have improved motivation and therefore performance of BALB/c mice in the current study compared to that of Graybeal et al. (2014). The impact of experimental design may influence the performance of BALB/c mice to a greater extent than more resilient strains such as C57BL/6 mice. For example, chronic mild stress has been shown to reduce spatial memory in conjunction with a reduction in hippocampal neurons and subregion thickness in BALB/c but not C57BL/6 mice (Palumbo et al., 2010). In addition, it has been shown that novelty-induced stress leads to a significantly greater increase in corticosterone concentrations in BALB/c compared to C57BL/6 mice (Brinks et al., 2007). Therefore, it should be expected that mouse strains will respond differently to experimental conditions and this needs to be considered when measuring cognitive abilities.

Other subtle changes that were made in the current study included reducing the ITI (from 15–20 s to 5 s) and increasing the number of trials per session (from 30 to 100 trials). These changes were made based on the observation of mice engaging in alternative behaviors between trials and even sleeping towards the end of the session. Once trial pace was increased, mice were able to start the next trial soon after consuming the reward and appeared to be less distracted between trials. From observation, the mice appear to develop a lapping or repetitive-type action moving from the touchscreen to the reward receptacle and back again. This may facilitate learning in some strains to greater extent than others. The increase in trial pace also facilitated the increase in trials completed per session. By performing more trials, the mouse gains greater experience each session and this was expected to improve the rate of task acquisition. There was also a benefit of gaining more data samples per session, which should reduce variability in measures such as reward latency but would also allow a greater number of stimulus manipulations to be used within a session. It should be noted that the maximum session duration was still 1 h, so this manipulation did not require additional testing time. In rats it was found that increasing the number of trials from 20 to 60 per session improved performance (Bussey et al., 2008). However, it was reported that performance in rats was improved when using long ITIs compared to short ITIs (5 s vs. 20 s). Rats and mice vary in their performance of operant behaviors and therefore this finding may be species-specific. Given the significant improvement observed in BALB/c performance, it is possible that there may even be strain-specific preference for long or shorter ITI periods. In this study, the tone presented for a correct response was removed as the reward pump can easily be heard within the chamber and functioned as an immediate auditory signal the mouse was correct. We noted that the tone was much louder and could be heard clearly from outside the sound-attenuating chambers. Although mice are likely to discriminate the tone generated in their chamber from others being tested simultaneously, the association may be unclear during initial training and could even be mildly aversive for an anxious strain. Therefore, it was removed from this study and was of no detriment to learning, as seen in C57BL/6 mice, but may have contributed to the improved task acquisition observed in BALB/c mice. These changes, as well as using a liquid reward, were made based on our previous observations to improve performance and learning. While the responses of C57BL/6 mice were comparable to other studies, the improvement in BALB/c performance compared to previous results was substantial. The optimal touchscreen protocols parameters for different mouse strains remains to be fully tested and they may be an important consideration when interpreting cognitive abilities.

One explanation proposed for the previous reports of poor performance in BALB/c mice has been reduced visual capabilities due to albinism (Yeritsyan et al., 2012). Graybeal et al. (2014) found that the albino A/J and FVB/NJ strains were also unable to acquire VD while the other pigmented 129S1/SvImJ and DBA/2J strains were able to learn both discrimination and reversal tasks. These results suggest visual deficits associated with albinism may interfere with VD learning using touchscreen images. It is generally agreed that albino strains have poorer visual acuity than pigmented strains (Prusky et al., 2002; Brown and Wong, 2007). On the rCPT touchscreen task albino CD1 mice failed to advance from visual detection to discrimination. They were then subsequently tested on a VD task with large stimuli, on which they still did not improve and it was suggested these results are due to poor perceptual ability (Kim et al., 2015). In rats, touchscreen VD studies have reported both equal (Bussey et al., 2008) or worse (Kumar et al., 2015) performance in albino Sprague-Dawley compared to pigmented Lister-Hooded rats. With mixed reports and the majority of touchscreen studies being conducted in pigmented rats and mice, it is unclear if and/or how albinism may restrict touchscreen task performance. However, the results of our study demonstrate that albinism in the BALB/c mouse is not a barrier to using visual stimuli and perhaps other factors may interfere with task acquisition.

In this study we have shown a substantially different outcome for BALB/c mice than previously reported and while we speculate minor changes to task design may have contributed to this finding, we did not directly compare task variations and therefore other explanations must also be considered. The mice were obtained from different suppliers, which may lead to differences in the genetic background, transport history and breeding/housing conditions. The results of the open field test and FST demonstrate that the cohorts of BALB/c and C57BL/6 mice used for this study had the strain-specific differences in locomotion and behavioral despair that have been documented elsewhere (Crawley et al., 1997; Belzung and Griebel, 2001; Tang et al., 2002; Groves et al., 2013). Furthermore, these behavioral tests were conducted after touchscreen testing had ceased, indicating the effects of long-term food-restriction, daily handling and cognitive training did not abolish these strain-specific characteristics. These findings are important as they demonstrate that the cohort of mice tested in this study did retain typical strain-specific phenotypes. Although there was a significant difference between strains in incorrect response latency, there were no differences in correct or reward latency. These results suggest disparities in touchscreen performance are unlikely to be due to motoric differences between the strains. Other factors that could alter the results include experimental differences, such as habituation to the room prior to testing, or different handling techniques could also contribute to altered behavioral performance. Even with care to directly replicate procedures, it has been shown that behavioral outcomes vary between laboratories (Crabbe et al., 1999). However, one of the advantages of using touchscreen operant chambers should be the reduction of experimenter effects and a more objective measurement of cognitive behavior across locations.

The majority of studies do not report data separated for images, however the images used in this study led to different acquisition rates in C57BL/6 mice during VD and in both strains during reversal. The “fan” and “marble” shaped stimuli were used in the previous BALB/c mice study (Graybeal et al., 2014), however a comparison of learning rates between images was not presented. Although the “fan” and “marble” combination are commonly used in mice, it is uncommon to see the results split for each image (Romberg et al., 2013). In an early study of touchscreen performance in mice, the “fan/marble” stimuli resulted in accuracy ranging from ~35 to 70% correct across eight individual mice on day 1, perhaps indicating individual preference or stimulus bias to these images (Bussey et al., 2001). Within the current study, responding to the four small circles appears to be favored and on reversal this preference was evident in both strains. Image salience differences may be used to investigate how top-down processing overcomes the innate bottom-up drive to respond to a particular image. Perhaps this battle was overcome easily in BALB/c mice during simple discrimination learning, however with the added cognitive load of reversal learning the discrepancy between choosing the sensory-driven vs. task-dependent image may become more apparent. Although it is suggested that stimulus pairs should minimize bias, these findings may be useful for altering cognitive load and task difficulty and may provide additional insights into learning and decision-making. A touchscreen study investigating cognitive performance in* Fmr1KO* mice, an animal model of Fragile-X syndrome, found that errors were increased within one stimulus group, but not the other, and only on the second reversal in a serial reversal task (Dickson et al., 2013). Image bias may increase overall performance variability, however splitting animals into stimulus groups could be useful for manipulating cognitive load in future studies. However, while studies continue to report pooled data rather than results split for each stimulus, it will be difficult to determine the extent of stimuli bias and the influence it has on touchscreen performance.

## Conclusion

Contrary to previous results, we found that BALB/c mice were not only capable of learning VD and reversal touchscreen protocols but their performance was superior to C57BL/6 mice. Subtle modifications were made to the standard protocols and different testing conditions may have led to these conflicting, but promising results. These findings suggest that testing conditions can have a significant strain-specific impact on cognitive performance in mice. Future studies should systematically determine the optimal touchscreen protocol parameters for mice with consideration for strain differences.

## Author Contributions

KMT and THJB conceived and designed the experiments. CGS and KMT performed the experiments. KMT analyzed the data. THJB contributed reagents/materials/analysis tools. KMT, CGS and THJB wrote the article.

## Funding

This work was funded by the National Health and Medical Research Council of Australia, Grant number APP1070081, and a University of Queensland Major Equipment and Infrastructure Grant MEI2014000118 to THJB. KMT was supported by the Queensland Government Smart Futures PhD Scholarship and an Australian Postgraduate Award. The funders had no role in study design; in the collection, analysis and interpretation of data; in the writing of the report; and in the decision to submit the article for publication.

## Conflict of Interest Statement

The authors declare that the research was conducted in the absence of any commercial or financial relationships that could be construed as a potential conflict of interest.
